# Assessing and Comparing Information Security in Swiss Hospitals

**DOI:** 10.2196/ijmr.2137

**Published:** 2012-11-07

**Authors:** Sarah Landolt, Jürg Hirschel, Thomas Schlienger, Walter Businger, Alex M Zbinden

**Affiliations:** 1 AAC InfotrayWinterthurSwitzerland; 2 Ypsomed AGBurgdorfSwitzerland; 3 TreeSolution Consulting GmbHHünenbergSwitzerland; 4 Engineering and Information TechnologyBern University of Applied SciencesBernSwitzerland; 5 Medical Technology CenterBern University of Applied SciencesBernSwitzerland

**Keywords:** information security, information protection, computer security standards, electronic health records organization & administration, hospital information systems, Switzerland.

## Abstract

**Background:**

Availability of information in hospitals is an important prerequisite for good service. Significant resources have been invested to improve the availability of information, but it is also vital that the security of this information can be guaranteed.

**Objective:**

The goal of this study was to assess information security in hospitals through a questionnaire based on the International Organization for Standardization (ISO) and the International Electrotechnical Commission (IEC) standard ISO/IEC 27002, evaluating *Information technology – Security techniques – Code of practice for information-security management*, with a special focus on the effect of the hospitals’ size and type.

**Methods:**

The survey, set up as a cross-sectional study, was conducted in January 2011. The chief information officers (CIOs) of 112 hospitals in German-speaking Switzerland were invited to participate. The online questionnaire was designed to be fast and easy to complete to maximize participation. To group the analyzed controls of the ISO/IEC standard 27002 in a meaningful way, a factor analysis was performed. A linear score from 0 (not implemented) to 3 (fully implemented) was introduced. The scores of the hospitals were then analyzed for significant differences in any of the factors with respect to size and type of hospital. The participating hospitals were offered a benchmark report about their status.

**Results:**

The 51 participating hospitals had an average score of 51.1% (range 30.6% - 81.9%) out of a possible 100% where all items in the questionnaire were fully implemented. Room for improvement could be identified, especially for the factors covering “process and quality management” (average score 1.3 ± 0.8 out of a maximum of 3) and “organization and risk management” (average score 1.3 ± 0.7 out of a maximum of 3). Private hospitals scored significantly higher than university hospitals in the implementation of “security zones” and “backup” (*P* = .008).

**Conclusions:**

Half (50.00%, 8588/17,177) of all assessed hospital beds in German-speaking Switzerland are in hospitals that have a score of 49% or less of the maximum possible score in information security. Patient data need to be better protected because of the data protection laws and because sensitive, personal data should be guaranteed confidentiality, integrity, and availability.

## Introduction

Information management, especially in emergency medicine, enhances the instantaneous and ubiquitous availability of digital patient records and can significantly improve clinical practice [1]. On the other hand, poor patient data security represents a major problem that must be addressed with more sophisticated hospital information technology (HIT) [2], but the protection of information represents a growing challenge [3]. For example, it is increasingly difficult to safeguard the integrity of digital radiology images and protect them from unauthorized manipulation [4]. Furthermore, the growing integration of complex hospital information systems [5], the widespread use of mobile devices [6], and the increasing amount of communication between health care providers require special attention regarding information security.

To implement an adequate information-security management system, it is first necessary to evaluate information security and assess its risks, and subsequently to find suitable measures to control risks and improve security measures [7]. TheInternational Organization for Standardization (ISO) and the International Electrotechnical Commission (IEC) have defined the international standards for information and data security (ISO/IEC 2700x, *Information technology – Security techniques*) that are widely accepted and can be used to evaluate levels of security [8]. The standards identify three main components of information security: confidentiality, availability, and integrity. They also describe requirements for an information-security management system (ISO/IEC 27001), a code of practice (ISO/IEC 27002) [9], implementation guidelines (ISO/IEC 27003), parameters to be measured (ISO/IEC 27004), and risk management (ISO/IEC 27005).

Education is an important component of successful management of information security [10]. To determine appropriate actions and education efforts, chief information officers (CIOs) need to know the status quo in their organization and have both a measuring tool and benchmark values at their disposal. However, no study has compared hospitals with respect to information security. This might be because information about the security level of an institution is delicate and might influence the hospital’s perceived trustworthiness or that assessing it might itself be a security threat. The lack of an effective benchmark tool for the assessment of the status quo of information security may be another explanation for the absence of such comparisons: The comprehensive and time-intensive character of commercially available tools, such as Verinice [11], rules out their use for a widescale comparison of hospitals.

Switzerland has a national implementation strategy for efficient and safe eHealth systems in which, for reasons of legal rights and acceptability, information security plays a central role [12]. The goal of this study is to evaluate the current status of information security in Swiss hospitals. As a first step, an ISO/IEC 27002-compliant tool that allows for both a rapid nationwide assessment of hospital security and the provision of benchmark data for CIOs was developed. By using this tool, the present investigation aims to evaluate information security focusing on differences between hospitals of different sizes and types (ie, private vs public hospitals and academic vs non-academic hospitals).

## Methods

### Questionnaire

The goal was to develop an online questionnaire that covered most chapters of the ISO/IEC standard 27002, *Information technology – Security techniques – Code of practice for information-security management*, and required less than 20 minutes to fill out. The online “EFS Survey” tool [13] was used to design and host the questionnaire. The questionnaire incorporated 24 parameters defined in the ISO/IEC 27002 standard, *Information technology – Security techniques – Code of practice for information-security management*, with some parameters combined into one question (see Appendix 1 for the complete set of questions with the corresponding chapters of the standard). All questions were identically structured and had four possible answers: (1) “unknown/not implemented,” (2) “partially implemented,” (3) “completely implemented,” and (4) “completely implemented and continuously monitored and improved.” The same layout and order of answers was used for all questions to reduce visual complexity. The questionnaire consisted of 19 screens, with 2 questions displayed on each screen. Questions regarding general parameters of the hospital, such as type of hospital, number of beds, number of full-time equivalents (FTEs) of job positions, total number of employees, number of FTEs in information technology (IT), total number of employees in IT, and number of computer workstations at the hospital, were assessed before the actual questionnaire. The questionnaire was reviewed by several national experts from the fields of medical informatics and information security not directly involved in the survey. To ensure technical functionality, the questionnaire was comprehensively tested by three test participants prior to its distribution.

The CIOs of all 112 hospitals in the German-speaking portion of Switzerland were informed via email about the planned study 6 months before its inception. They were also informed that the study would be conducted by students of a Master of Advanced Studies in Medical Informatics at the Berne University of Applied Science. The survey was announced a second time in a personal letter [14] and, 2 weeks later, a third time via a personal email that contained a hyperlink with a personal key. Participants were informed in the correspondence that the survey should take a maximum of 15 to 20 minutes to complete and that confidential treatment of data was guaranteed.

To encourage timely responses, a genographic test from National Geographic [15] was offered as draw prize to one of the first 50 participants. Furthermore, the participants were ensured a detailed benchmark analysis. Participants were asked to respond within 2 months. To improve the response rate, two reminder emails were sent out 10 days after this 2-month period and again 1 week later.

The participants gave informed consent by affirming the opening question of the questionnaire: “Do you agree to participate in our survey? And do you give your consent that the data may be published in an anonymized form?”

There was a unique key included in the individual hyperlinks sent out to participants to access the survey tool. The key was logged by the tool and exported with the data; therefore, duplicate entries from the same user were precluded. The tool was configured to continue only after a question was completed. A back button allowed for corrections.

Data with personal information were stored in encrypted form when exported from the survey tool. Persons involved in the statistical evaluation were blinded and worked with anonymized data. A monitoring group was in charge of protecting the data and the interests of the participants. All students involved in the investigation were required to sign a confidentiality agreement.

### Statistical Analysis

Scores were introduced to perform a statistical analysis of the data collected. A linear score from 0 (answer 1) to 3 (answer 4) was used, as shown in Table 1. The higher a hospital’s overall score, the more sophisticated its data security management.

**Table 1:** The four possible answers to questionnaire items and the assigned score points.

Answer	Score points
1. Unknown, not implemented	0
2. Known, partially implemented	1
3. Completely implemented	2
4. Completely implemented, under continuous improvement	3

Hospitals were classified into (1) academic (university) hospitals with a research mandate from the state, (2) non-academic public hospitals with an emergency ward, (3) rehabilitation clinics, and (4) private hospitals. Furthermore, the hospitals were split into two groups based on hospital size (ie, hospitals with > 150 beds and hospitals with ≤ 150 beds.

For data reduction, a factor analysis with varimax rotation and Kaiser normalization using SPSS version 15 (SPSS Inc, Chicago, IL, USA) was applied to group-related questions into independent factors.The Kaiser normalization eliminated all components with eigenvalues under 1.0, thus extracting 7 reasonable factors. No further cutoff criteria for determining the optimal number of factors were explored.

Since normal distribution could not be shown using a Shapiro-Wilk test, a two-way non-parametric analysis of variance (Friedman test) was performed for both the type of risk factors determined by the factor analysis and the group of hospitals. The difference between hospital types was then determined using pairwise testing with Bonferroni’s corrections. Subsequently, the influence of each of the 7 risk factors on the differentiation between hospital types was calculated using a non-parametric one-way analysis of variance (Kruskal-Wallis test). The effect of hospital size (number of beds) was determined using a Friedman test, also taking into account the 7 risk factors.

## Results

### Questionnaire

Of the 112 CIOs invited to participate in the survey, 69 (61.6%) responded. Of these, 7 did not give informed consent, 9 aborted the questionnaire while answering the general questions about the hospital, and 2 aborted the questionnaire while answering the questions about information security. In Table 2, “responded,” “participated,” and “completed” indicate that the survey page was visited, that the informed consent page was filled out, and that the questionnaire was fully completed, respectively. Thus, there was a 90% (62/69) participation rate and a 74% (51/69) completion rate [16]. Only the 51 completed datasets were used for further analysis.

**Table 2:** Analysis of the number and percentage of returned questionnaires with respect to hospital type and hospital size.

**Group**		**Invited**		**Responded**		**Participated**		**Completed**	
		n	%	n	%	n	%	n	%
**Hospital type (total)**		112	100%	69	62%	62	90%	51	74%
	University hospital	11	100%	9	82%	6	67%	4	44%
	Public hospital	54	100%	39	72%	36	92%	29	74%
	Rehabilitation clinic	13	100%	7	54%	7	100%	6	86%
	Private hospital	34	100%	14	41%	13	93%	12	86%
**Hospital size (total)**		112	100%	69	62%	62	90%	51	74%
	≤ 150 beds	45	100%	20	44%	18	90%	16	80%
	> 150 beds	67	100%	49	73%	44	90%	35	71%

Of the 51 hospitals in which a CIO had completed the questionnaire, 4 were university hospitals, 29 were public hospitals, 6 were rehabilitation clinics, and 12 were private hospitals.The hospitals which completed the questionnaire had total scores ranging from 30.6% to 81.9% out of a maximum score of 100%. These scores are presented in Table 3 for the two hospital sizes and for the four hospital types.

**Figure 1:**
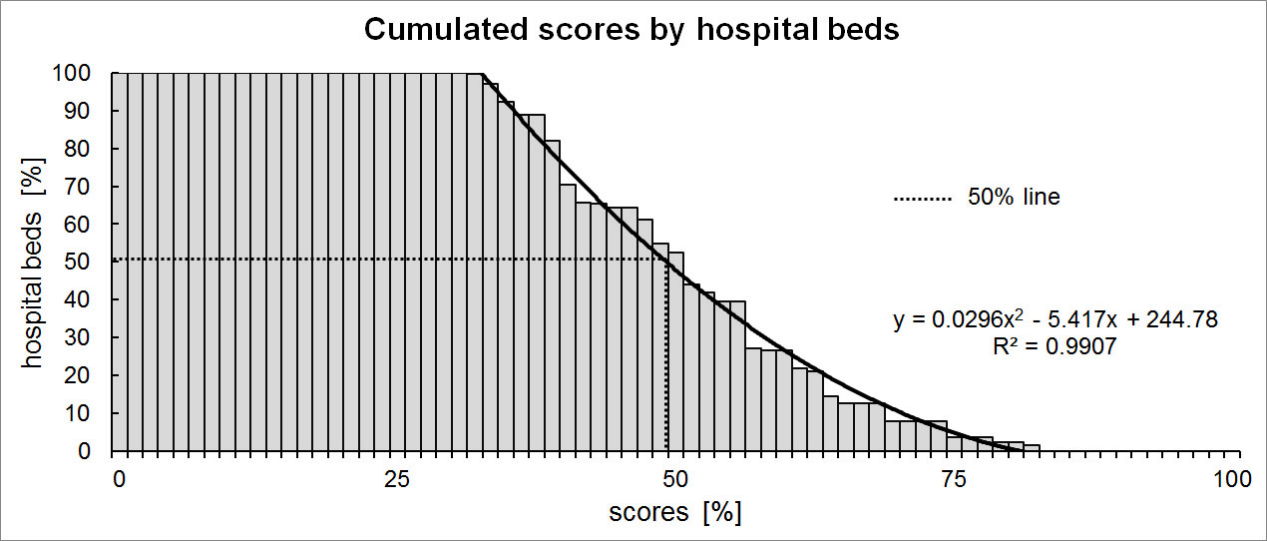
Cumulated scores by hospital beds.

To visualize the overall distribution of information security for hospitals in German-speaking Switzerland, each hospital’s individual score was calculated as a percentage of the maximum score. These percentages are shown in Figure 1 as functions of the number of beds in a hospital. Additionally, a least squares regression curve was laid over the cumulated scores. The curve characterizes the distribution of information security per hospital bed. The 50% line shows that, according to the regression curve, 50% of all hospital beds reached a score of 49.2% or less of the maximum score.

The factor analysis extracted 7 factors, explaining 70% of the total variance. The questions were assigned to the factor with the highest correlation (Table 4).

The grouping of the questions into factors gave interesting insights into their relationship and made it possible to assign a group term to each of the 7 groups of questions (Table 5).

**Table 3:** Scores for each hospital type and for the different hospital sizes.

Group		Average score		Minimum score		Maximum score	
		Mean	%	Mean	%	Mean	%
**Hospital type (total)**		36.8	51.1%	22	30.6%	59	81.9%
	University hospital	32.8	45.5%	24	33.3%	40	55.6%
	Public hospital	36.4	50.6%	22	30.6%	59	81.9%
	Rehabilitation clinic	35.2	48.8%	23	31.9%	56	77.8%
	Private hospital	39.9	55.4%	30	41.7%	53	73.6%
**Hospital size (total)**		36.8	51.1%	22	30.6%	59	81.9%
	≤ 150 beds	36.8	51.1%	22	30.6%	58	80.6%
	> 150 beds	36.8	51.2%	23	31.9%	59	81.9%

**Table 4:** Results of the factor analysis (rotated component matrix).

								
**1. Process and quality management**								
	5. Classification of information	0.79^a^	–0.08	0.19	–0.07	–0.05	0.01	0.27
	7. Awareness and end-user training	0.45^a^	0.22	0.25	0.25	0.21	0.27	–0.11
	9. Documented business processes	0.57^a^	–0.06	–0.01	0.36	–0.11	0.11	0.13
	21. Security incidents reporting	0.70^a^	0.30	0.19	0.01	0.11	0.09	–0.08
	22. Learning from incidents	0.81^a^	0.23	0.05	0.26	0.15	0.13	–0.11
	23. Ensuring hospital business continuity	0.69^a^	0.10	0.21	–0.23	0.12	–0.09	0.42
**2. Access control and procurement**								
	13. Policies for handling mobile storage devices	0.02	0.54^a^	0.14	0.18	0.14	0.43	–0.01
	15. User management and access rights	0.13	0.77^a^	0.06	0.10	–0.10	0.20	0.17
	16. Remote access control	–0.02	0.82^a^	0.17	–0.16	0.30	0.09	–0.03
	18. Secure procurement	0.28	0.69^a^	0.02	0.27	0.21	–0.01	0.19
**3. Organization and risk management**								
	1. Security-risk analysis	0.38	0.07	0.58^a^	–0.24	–0.09	0.00	0.03
	2. Information-security policies	0.09	0.08	0.72^a^	0.42	0.21	0.10	0.01
	3. Management commitment	0.10	0.09	0.88^a^	0.08	0.01	0.21	0.00
	4. IT inventory and data ownership	0.38	0.17	0.50^a^	0.47	–0.31	–0.06	–0.08
**4. Control and monitoring**								
	6. Employment-contract rules	0.22	0.07	0.07	0.61^a^	0.02	0.49	–0.13
	14. Monitoring	–0.10	0.13	0.14	0.74^a^	0.22	–0.05	0.26
	17. System-login security	0.34	0.44	–0.13	0.46^a^	0.05	0.12	0.36
**5. Attack protection**								
	11. Malware protection	0.35	0.36	–0.30	0.16	0.56^a^	0.07	–0.09
	20. Patch management	–0.30	0.20	–0.09	0.15	0.64^a^	0.25	0.17
	24. Security assessments	0.30	0.06	0.40	–0.04	0.65^a^	0.05	–0.09
**6. Encryption and staging**								
	10. Staging (separation of development, test, and productive environment)	0.42	0.27	0.13	0.04	0.17	0.57^a^	–0.14
	19. Encryption of mobile data	–0.02	0.15	0.11	–0.01	0.11	0.84^a^	0.18
**7. Backup and security zones**								
	8. Security zones	0.34	0.22	0.10	0.20	0.47	0.29	0.50^a^
	12. Backup	0.06	0.12	–0.04	0.12	–0.02	0.03	0.86^a^

^a^highest correlation value per question.

**Table 5:** Terms given to the seven factor groups of questions.

Factor	Term
Factor 1	Process and quality management
Factor 2	Access control and procurement
Factor 3	Organization and risk management
Factor 4	Control and monitoring
Factor 5	Attack protection
Factor 6	Encryption and staging
Factor 7	Backup and security zones

**Figure 2:**
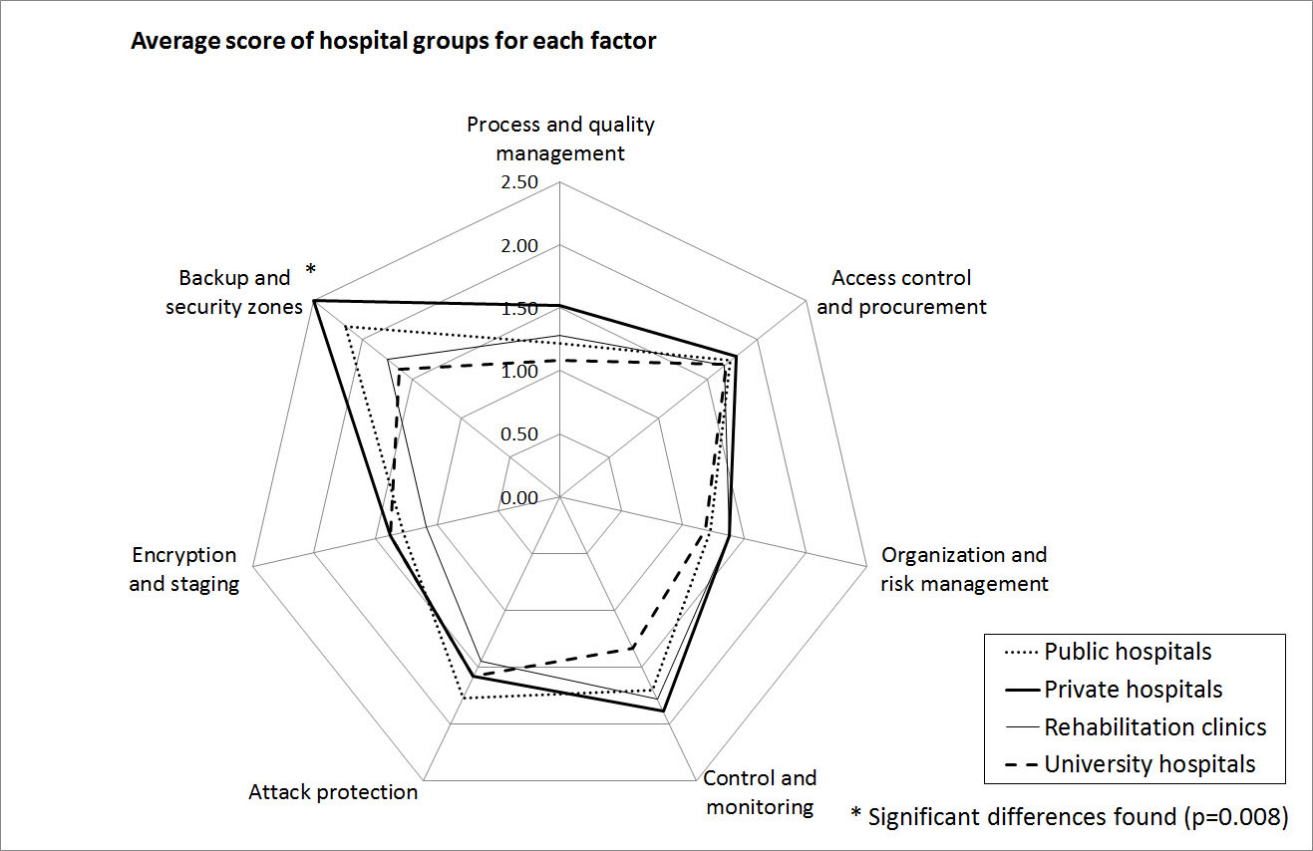
Scores by hospital groups.

**Figure 3:**
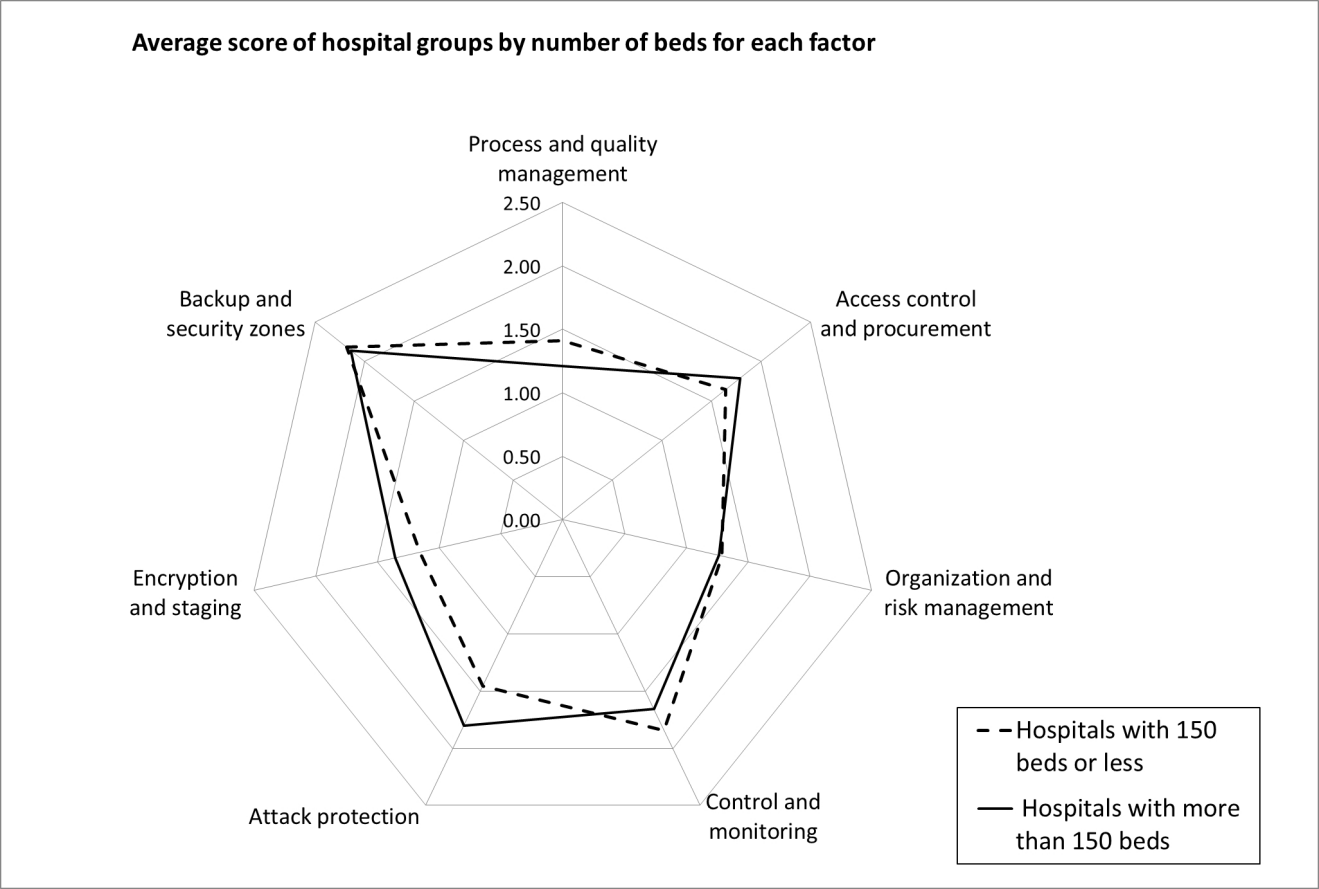
Scores by hospital size.

The null hypothesis that all hospital types reach the same scores could be rejected (*P* < .05) on the basis of the Friedman test for all 28 groups (7 risk-factor groups and 4 types of hospitals). With the Kruskal-Wallis test, a significant difference (*P* < .05) in factor 7 (backup and security zones) between hospital types was found, with university hospitals ranking lowest and private hospitals highest (Figure 2). Using (conservative) pairwise testing and Bonferroni’s correction, however, no significant difference was found (*P* = .02 which is greater than .05/7 = .0071). No significant effect was observed with respect to hospital size (Figure 3).

## Discussion

### Main Results

In this investigation, a comprehensive, but efficient and rapid, method to survey information security in institutions was introduced and successfully applied in 51 Swiss hospitals. Half (50.00%, 8588/17,177) of all hospital beds had a score of less than 49.2% of the maximum possible score (100%). In other words, a patient in one of these hospitals runs a 50% risk that he or she will lie in a hospital bed for which information security scores only reach 49%. Furthermore, university hospitals had lower scores for basic security features than private hospitals, although this difference does not reach statistical significance when conservative testing procedures are applied.

### Methods

The lack of tools to quickly and inexpensively assess the information security of large numbers of hospitals led us to develop an effective and comprehensive survey tool. Only the 24 most-pertinent parameters of the total 133 parameters in the ISO/IEC 27002 standard were included to keep the resulting questionnaire manageable in a reasonable amount of time and to restrict the amount of data generated. This was possible by combining several subchapters into one parameter and selecting questions especially relevant to hospitals.

Tools, such as the one presented here, will become increasingly important as more and more countries need to address issues of information security in their health care systems.

### Questionnaire

Only the data of hospitals that fully completed the questionnaire were analyzed. However, a selection bias may have influenced participation: Hospitals envisaging a potential for improvement in their security management may have been more willing to fill out the questionnaire to receive free advice through our benchmark report. On the other hand, hospitals apprehensive of a bad ranking might have refrained from answering. Unfortunately, this bias cannot be verified retrospectively.

Only 44% (20/45) of the smaller hospitals responded to the survey, compared to 73% (49/67) of the larger hospitals (Table 2). It is possible that smaller hospitals have fewer IT resources and, therefore, did not take the time to fill out our questionnaire. However, it should be noted that larger hospitals aborted the questionnaire more frequently. Fewer university hospitals completed the questionnaire than other hospitals (Table 2). The number of invited university hospitals may appear misleadingly high, as the German-speaking part of Switzerland only has three university hospitals. However, several university hospitals have subunits (eg, children’s hospitals) with completely or partially independent IT structures. Fortunately, for each university hospital, exactly one eligible person representing the entire institution filled in the questionnaire. It is likely that these institutions appointed someone to respond, which also explains the frequency of aborted questionnaires.

Whether the responders filled out the questionnaire truthfully, whether they portrayed information security as more sophisticated than it actually is, or whether some respondents even understated their hospital’s performance to be able to apply for more funds for their department remains unclear. These questions can only be explored with an on-site investigation.

### Data Processing

Meaningful groups of security items were formed using factor analysis. The items of the first 5 factors dealt with similar topics. The final 2 factors, however, mixed different topics. This led to a decrease of eigenvalues and of the explanatory power of higher factor numbers as a consequence because of the very nature of factor analysis. Although all items in factor 6 (encryption and staging) showed an unambiguous high correlation with their factor, the mapping of question 8 about “security zones” to factor 7 (backup and security zones) was less straightforward because this question also showed high correlation with factor 5 (attack protection) to which it might also have been attributed based on its content. However, the authors decided to base factor attribution on the highest correlation and accepted the automated mapping suggested by the factor analysis.

### Limitations

Switzerland is a small country with four different linguistic regions. Because only hospitals in the German-speaking part of Switzerland were included, the number of hospitals surveyed was relatively low. It would be interesting to perform the study in a larger, more uniform country to be able to work with larger numbers.

### Risks for Patients

Of the 133 controls in the ISO/IEC 27002, *Information technology – Security techniques – Code of practice for information-security management*, the 24 that referred to issues of basic security were selected for our questionnaire.

Secure information processing in hospitals, such as preventing the loss or the (conscious or unintentional) manipulation of data, is crucial for patients’ health. Moreover, patient health data is protected by law in Switzerland: All patient data must be stored, transmitted, and processed in a secure way that ensures confidentiality and integrity. Our results showed that only 50% of the hospital beds reach 50% of the maximum security score, implying a substantial need for improvement in many of the controls surveyed.

### Recommendations for Hospitals

To address the most evident risks found in Swiss hospitals, we recommend considering the following points. We limit ourselves to the 8 questions with the lowest scores (see Appendix 1 for questions and for the reference to the ISO/IEC 27002 section [9]):

1. Risk assessments should be conducted regularly to identify, quantify, and prioritize risks (Question 1). The results should guide and determine the appropriate management action and help to prioritize controls to protect against these risks.
2. Information-security policies, standards, and guidelines should be created, approved by management, published, and communicated to all employees and relevant external parties (Question 2). These documents define how security is managed within the hospital. They should be regularly reviewed to ensure their suitability, adequacy, and effectiveness.
3. Management should actively support security within the hospital (Question 3). Administrative management and medical management should support security through clear direction, demonstrated commitment, explicit assignment, and acknowledgment of information-security responsibilities. The support of the management is one of the most important pillars of a strong information-security culture.
4. Information classification should be implemented in the hospitals (Question 5). Information has different values and may be subject to different regulations. Knowing the value, sensitivity, and importance of hospital data allows for prioritizing the protection measures.
5. A policy on the use of cryptographic controls should be developed (Question 19). Today an increasing number of internal and external systems exchange data with each other over the Internet. These data are subject to data protection law and have to be protected. Cryptographic controls allow a secure data exchange and guarantees the integrity and authenticity of the hospital data.
6. Responsibilities and procedures should be established to handle information-security incidents effectively once they have been reported (Question 22). A process of continual improvement should be applied to learn from such events.
7. A business continuity plan should be developed and maintained for business continuity throughout the hospital (Question 23). Business continuity protects critical business processes from the effects of major failures of information systems or disasters. It is especially important for the critical infrastructure of hospitals.
8. The security of the hospital information systems should be reviewed regularly (Question 24). Different approaches exist to review the compliance of information processing with policies and standards, such as baseline audits, penetration tests, or vulnerability scans. Such reviews will reveal weaknesses and allow for prioritizing the protection measures.

Implementing these measures will close the most important information security gaps in Swiss hospitals. They also lay the foundation for further security optimizations.

### Conclusions

In this paper, a comprehensive and efficient survey tool to obtain meaningful data concerning information security was introduced. Applied to assess information security in hospitals within the German-speaking section of Switzerland, it revealed surprisingly low security scores, especially for basic security issues. These results raise serious questions as to whether Swiss hospitals meet their patients’ expectations and the country’s legal requirements with regard to the level of information security they can guarantee. Our survey identified an urgent need for action to improve information security in hospitals, independent of their size and type.

In the future, the need for secure information handling in hospitals will increase greatly because of increased IT usage and digitalization in the health care sector. Information must also be protected from cyber threats that are increasing in number and sophistication. In the future, we will see more cyber threats that will directly attack industrial plants or a country’s or region’s critical infrastructures [17,18]. Hospitals are part of this critical infrastructure of a country; therefore, they must be protected from such information security breaches.

## Multimedia Appendix 1

Questions assessed in the online survey.

## Abbreviations

CIO: chief information officer
FTE: full-time equivalents
IEC: International Electrotechnical Commission
ISO: International Organization for Standardization
ISO/IEC 27002 standard: Information technology – Security techniques – Code of practice for information security management
