# Supplementary material for: Assessing and Comparing Information Security in Swiss Hospitals
Source: Interact J Med Res. 2012 Nov 7;1(2):e11. doi: 10.2196/ijmr.2137 (PMC3626122; doi:10.2196/ijmr.2137)
Supplement: Supplementary file 1 [file ijmr_v1i2e11_app1.pdf]

Appendix

Appendix 1: Questions assessed in the online survey

#	*Question in the survey*	*Rank*	*Average score (0=lowest, 3=highest)*	*Standard deviation*	*Reference to chapters of the norm ISO 27002[9]*
**1**	Do you regularly conduct risk assessments (or risk analyses) to account for changes in security needs and the risk situation?	20	1.24	0.65	4.1; (4.2); (6.2);(10.2); (11.7)
**2**	Do you establish and distribute guidelines for information security and review them regularly or at defined points in time?	19	1.25	0.56	5.1; (10.8); (11.7); (15.1)
**3**	Is management involved in information security (e.g., approval of information security guidelines, monitoring their effectiveness, supplying the resources, defining responsibilities)?	21	1.12	0.68	6.1
**4**	Is an inventory of data, data files, databases, software and hardware of the hospital taken and assigned to their owners?	11	1.49	0.78	7.1
**5**	Is hospital data classified?	24	0.98	0.73	7.2
**6**	Are staff members obliged to observe the rules concerning information security?	7	1.71	0.78	8.1
**7**	Are staff members trained in and made aware of the risks involved when handling and processing data?	12	1.41	0.85	8.2; (11.3)
**8**	Are there defined security zones (e.g., server rooms, back-up safe, etc.)?	5	1.90	0.81	9.1
**9**	Are business processes documented in detail?	16	1.33	0.79	10.1
**10**	Are development, testing and production environments separated?	10	1.51	0.86	10.1.4
**11**	How is malware protection organized?	2	2.29	0.58	10.4
**12**	Are software backups and data backups created regularly?	1	2.41	0.57	10.5
**13**	Do you have guidelines concerning the handling of mobile storage devices?	15	1.35	0.82	10.7
**14**	Are logs and protocols implemented to document user activity, errors and information security	12	1.41	0.54	10.10;

	incidents?				(12.5)
**15**	Is a process established to create, change and delete users to grant, change and withdraw access to information systems?	6	1.86	0.80	11.2; (8.3); (11.1)
**16**	Are rules and measures defined and implemented to regulate remote access to the hospital network?	3	2.08	0.82	11.4; (10.8)
**17**	Is each operating system protected with a username and a password?	4	2.06	0.68	11.5
**18**	Are requirements for information security taken into account when procuring new information systems?	9	1.65	0.74	12.1; (10.3)
**19**	Is information on mobile storage devices protected in an adequate way through encryption?	22	1.06	0.76	12.3
**20**	Is information about vulnerabilities in the information systems provided and rated in a timely manner, and are suitable measures taken? (Patch management)	8	1.69	0.79	12.6; (12.4)
**21**	Do users have the possibility to report information-security incidents through a reporting process?	14	1.37	0.85	13.1
**22**	Are necessary conclusions drawn from information security incidents?	17	1.31	0.71	13.2
**23**	To what extent are plans in place that guarantee the regular operation of the hospital in cases of breakdown or loss of data, software, hardware and services (e.g. in the case of a catastrophe)?	18	1.29	0.58	14.1; (9.2)
**24**	To what extent are security assessments, penetration tests and vulnerability scannings of the systems carried out?	23	1.04	0.85	15.2
